# Factors Influencing Abortion Decision-Making of Adolescents and Young Women: A Narrative Scoping Review

**DOI:** 10.3390/ijerph21030288

**Published:** 2024-03-01

**Authors:** Yui Koiwa, Eri Shishido, Shigeko Horiuchi

**Affiliations:** 1 Makita General Hospital, Nishikamata, Ota-ku, Tokyo 144-8501, Japan; 19mw301@slcn.ac.jp; 2 Department of Midwifery, Graduate School of Nursing Science, St. Luke’s International University, Akashicho, Chuo-ku, Tokyo 104-0044, Japan; eri-shishido@slcn.ac.jp

**Keywords:** adolescent pregnancy, young women, abortion, decision-making, factor, qualitative research

## Abstract

Introduction: Globally, about half of all induced abortions have been estimated to be unsafe, which results in 13% of maternal deaths yearly. Of these induced abortions, 41% of unsafe abortions have been reported in young women who are dependent on their parents for their livelihood. They are often left in a vulnerable position and may have difficulty in making a decision regarding abortion. This study aimed to (1) characterize and map factors that influence abortion decision-making of adolescents and young women, and (2) identify the care and support that they need in their decision-making process. Methods: We conducted a scoping review following the JBI method and PRISMA-ScR checklist. We comprehensively searched MEDLINE (PubMed), Embase, Cochrane Library, CINAHL, and PsycInfo, and hand searched publications in the Google Scholar database between November 2021 and October 2023. The search included all English language qualitative and mixed methods research articles published on the database up to October 2023 that included participants aged 10–24 years. The CASP checklist was used as a guide for the qualitative analysis. NVivo was used to synthesize the findings. Results: There were 18 studies from 14 countries (N = 1543 young women) that met the inclusion criteria. Three domains and eleven categories were included as follows: personal (desire for self-realization and unwanted pregnancy), interpersonal (parental impact, reaction of partner, roles of peers and friends, existence of own child, and lack of support), and social circumstances (sexual crime, financial problem, limitation of choice, and underutilized healthcare services). Decision-making factors regarding abortions were also found across all three domains. Conclusion: The abortion decision-making of young women is influenced by various external factors regardless of country. Parents are especially influential and tend to force their daughters to make a decision. Young women experienced suffering, frustration, and lack of autonomy in making decisions based on their preference. This emphasizes the importance of autonomous decision-making. In this regard, healthcare services should be used. However, there are barriers to accessing these services. To improve such access, the following are required: staff training to provide adolescent and youth-friendly health services, counseling based on women’s needs, counseling including the parents or guardians that is confidential and ethical, promotion of decision aids, and affordable accessible care.

## 1. Background

Globally, there are approximately 73 million induced abortions each year [1]. In Africa, 97% of all abortions were reported to be unsafe [2]. Between 2010 and 2014, the World Health Organization (WHO) estimated that 45% of all abortions were unsafe [1]. Among these 45% unsafe abortions [1], 15% were from adolescents aged 15–19 years, and 41% were from young women aged 20–24 years [3]. Unfortunately, unsafe abortions result in 13% of maternal deaths yearly [4].

The WHO defines an unsafe abortion as one that involves an unintended pregnancy and is performed by a person who lacks the appropriate skills or who carries out the abortion in an environment lacking sterility and medical asepsis, necessary equipment, evidenced-based interventions, and necessary aftercare [4]. Grimes et al. [5] reported that unsafe abortions can include traditional methods such as (1) the use of oral medications (e.g., toxic solutions, teas and herbal remedies, drugs), (2) treatments placed in the vagina or cervix, (3) intramuscular injections, (4) foreign bodies placed into the uterus through the cervix, (5) enemas, and (6) trauma. Several studies from developing countries similarly reported the use of traditional methods by unskilled providers for terminating unwanted pregnancies, resulting in unsafe abortions [6,7,8]. 

Even though 38 million adolescents reportedly need contraceptives in developing countries to avoid unintended pregnancy, the majority (60%) do not use them because of complex underlying factors [9]. Adolescent pregnancy carries with it a high risk of obstetric complications [10,11]. In fact, maternal factors are the second leading cause of death among adolescents aged 15–19 globally, with little change in the ranking since 2000 [12]. Yakubu et al. [13] described various factors influencing adolescent pregnancy in sub-Saharan Africa, which include sociocultural, environmental, and economic factors (e.g., unequal gender power relations, coercive sexual relations, poverty, lack of knowledge, nonusage of contraceptives, early sexual behavior). Individual and health service-related factors have also been identified. However unintended pregnancy and abortion are common experiences shared by many women globally, regardless of country, income group, and legal status [14]. Pregnant adolescents not only face obstetric complications but also have greatly changed life prospects. This is because adolescent pregnancy and childbearing often lead to dropping out of school. Consequently, the resulting lack of education inhibits their ability to build self-esteem, improve their status in the family and community, and have a say in decisions that affect their lives [15], which include career loss and the subsequent financial distress. Thus, unintended adolescent pregnancy may lead to various adverse physical, social, and economic outcomes [9,16]. These environments surrounding adolescent girls put them at risk. Importantly, unintended pregnancies can lead adolescents to choose unsafe abortion.

Shah and Ahman [17] reported that the rates of abortions are about the same regardless of country or legal restrictions. One consideration for women in seeking an unsafe abortion is whether abortion is legally permitted. Bearak et al. [14] found that the abortion rates from 2010–2014 did not substantially differ between countries where abortion was legally available compared with countries where abortion was restricted. The main difference was in safety. These social or legal barriers may influence women to seek unsafe abortions, or make them hesitant to seek urgent care for complications of unsafe abortions [4,17]. 

Incidentally, adolescents are more likely to have access to unsafe abortions and delay seeking care for complications after unsafe abortions than adults [18,19]. Adults and adolescents differ in terms of care-seeking behavior such that adolescents tend to (1) delay seeking an abortion, (2) seek an abortion from traditional providers or unskilled providers, (3) use dangerous methods, (4) have complications, (5) delay seeking help even after complications, (6) lack transportation, (7) lack of awareness of where to seek help, (8) disregard cost considerations, and (9) have poor perception of health workers’ attitudes [20]. These circumstances will most likely endanger adolescents when seeking an unsafe abortion. 

The decision to have an abortion can be difficult, especially for adolescents and young women who are in a vulnerable position of having to depend on their parents or other people for their livelihood. Although there has been ongoing qualitative research about abortion decision-making of adolescents and young women, to our knowledge, the body of the research literature has not yet been characterized or mapped. According to Arksey and O’Malley [21], a scoping study might aim to rapidly map the key concepts underpinning a research area and the main sources and types of evidence available.

Thus, a narrative scoping review of such qualitative or mixed methods research articles is urgently needed to validate and emphasize the crucial need for support of adolescents and young women in terms of abortion decision-making.

### 1.1. Research Question

(1) What are the factors that influence the abortion decision-making process of adolescents and young women?

(2) What care and support do adolescents and young women need in their abortion decision-making process?

### 1.2. Objectives 

This study aims to (1) characterize and map the factors that influence the abortion decision-making process of adolescents and young women, and to (2) identify the care and support that they need in their decision-making process.

## 2. Methods

This study used the scoping review framework by Arksey and O’Malley [21]. A scoping review is a type of knowledge synthesis that engages a systematic approach to map evidence on a topic and identify the main concepts, theories, sources, and knowledge gaps [22]. Moreover, scoping reviews may also be helpful precursors to systematic reviews [21,23]. Hence, this study used a scoping review that should have great utility for synthesizing and mapping extant research evidence. 

This scoping review adhered to the JBI guideline and the Preferred Reporting Items for Systematic reviews and Meta-Analyses extension for Scoping Reviews (PRISMA-ScR) checklist [22]. The Critical Appraisal Skills Programme (CASP) checklist was used for qualitative analysis of this study [24]. 

### 2.1. Inclusion Criteria

Articles were included if they met the following criteria that were based on the questions using the Population, Concept, and Context (PCC) framework provided by the JBI procedure [25]. The inclusion criteria were as follows: (1) adolescents and young women aged 10–24 years, (2) adolescents and young women who reported their attitude and decision-making process about abortion, (3) original and qualitative or mixed methods studies, and (4) English articles. All articles were published on the database up to October 2023 (Table 1).

#### Search Strategy

We comprehensively searched MEDLINE (PubMed), Embase, Cochrane Library, CINAHL, and PsycInfo, and hand searched publications in the Google Scholar database between November 2021 and October 2023. The search included all English language qualitative and mixed methods research published on the database up to October 2023, and included participants aged 10–24 years. The PCC framework guided the development of the keywords. An additional hand search for reference lists searches identified other new articles. An example of the complete search strategy for MEDLINE (PubMed) is listed in Appendix A.

### 2.2. Study Selection

**Figure 1:**
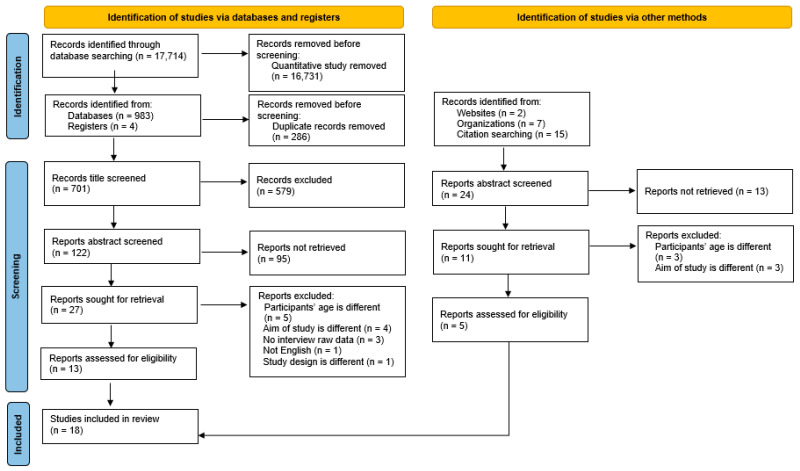
PRISMA 2020 flow diagram for new systematic reviews, which included searches of databases, registers, and other sources [27]. For more information, visit: http://www.prisma-statement.org/ (accessed on 23 January 2024).

There were 17,714 articles identified through database searching. Quantitative studies were removed, leaving 987 articles from the databases and registers. There were 24 articles retrieved from the other searches. After duplicates were removed, 725 studies were retained. An additional 579 records were removed following the review of titles. Of those, 108 articles were excluded following the review of the abstract screening based on the inclusion criteria. Then, 38 full-text articles were read and 20 articles were excluded (for exclusion reasons, see Appendix B). Seven papers were excluded because the target population was over the age of 24 years; five papers did not meet the inclusion criteria for the aim of this study; three papers had no raw data; and one paper was not in English. The process of selection is shown in the PRISMA flow diagram (Figure 1). Two independent reviewers evaluated and discussed the qualifications of the articles for inclusion. If disagreements could not be resolved through discussion, a third reviewer made the decision regarding the selection. Three studies included individuals 25 years and older. We were able to identify data by age and thus excluded participants 25 years and older. Finally, 18 studies were included in the qualitative synthesis.

### 2.3. Data Extraction

The articles were abstracted in terms of their publication details: authors, year, title, country, aim, study design, population, data collection methods, and key findings. Thereafter, data were imported into NVivo windows software, which extracted concepts by selecting, coding, and creating nodes (files) representing key concepts. Thematic analysis adopted qualitative content analysis by Elo and Kyngäs [28]. The lead researcher (YK) read the articles several times to understand the participants’ views and developed code. They subsequently discussed the initial data code with other coresearchers (SH and ES). The subcategories were classified based on their similarities, and the categories were developed from the grouped subcategories. And then they developed the domain from the categories. This analysis allowed for the exploration of the data by identifying (1) different themes in the environment surrounding adolescents and young women, (2) factors influencing abortion decision-making, and (3) perspectives toward abortion.

### 2.4. Quality Assessment

The research team consist of three reviewers (YK, SH, and ES) who independently assessed the quality of the articles using the CASP checklist [24]. The CASP checklist is a tool for systematically screening manuscripts through the enquiry and critique of this study’s validity, results, and research contributions. Quality assessment was undertaken and independently verified. There were no studies that were excluded.

## 3. Results

Eighteen studies meeting the inclusion criteria were included in this scoping review and are summarized in Table 2. The research studies were conducted in 14 countries: England (*n* = 3), South Africa (*n* = 2), Ghana (*n* = 2), Australia (*n* = 1), Brazil (*n* = 1), Burkina Faso (*n* = 1), Malawi (*n* = 1), Mexico (*n* = 1), Mozambique (*n* = 2), India (*n* = 1), Ethiopia, Malawi and Zambia (*n* = 1), Sweden (*n* = 1), and the United States of America (*n* = 1).

The studies were published from 2002 to 2023 and included 1543 young women. There were 16 qualitative studies, 2 of which involved secondary analyses [29,30,31,32,33,34,35,37,38,39,40,41,42,43,44,46], and there were 2 mixed methods studies [36,45]. There were 13 studies where all participants had abortion experiences and explored abortion decision-making [29,30,31,32,34,35,37,38,39,40,42,43,45]. 

Four studies included mixed participants who continued their pregnancy or had abortion experiences or never had pregnancies, and stakeholders who explored their choice and decision-making process regarding pregnancy outcomes [33,36,41,46]. There was one study involving a young migrant and refugee people’s views on unintended pregnancy and abortion [44].

### 3.1. Categories from the Data

Analysis of the included studies obtained three main domains of reasons for seeking an abortion: personal, interpersonal, and social circumstances. Decision-making factors regarding abortions were also found across all three domains.

Data extraction yielded 11 categories nested within the three domains: personal (desire for self-realization and unwanted pregnancy), interpersonal (parental impact, reaction of partner, roles of peers and friends, existence of own child, and lack of support), and social circumstances (sexual crime, financial problem, limitation of choice, and underutilized healthcare services). Table 2 shows a summary of the findings according to the three domains of the framework (influence abortion decision-making of adolescents and young women) and their associated categories and subcategories.

### 3.2. Personal

Two categories were included under this domain: desire for self-realization and unwanted pregnancy. They are described below with their associated subcategories.

#### 3.2.1. Desire for Self-Realization

Sixteen articles described a desire for self-realization, which had two subcategories: actualized her intentions and never give up education and career. 

Young women who are dependent on their parents for their livelihood are often left in a vulnerable position. However, the desire to have autonomy and make decisions for themselves has been stated in many articles. These reasons can be summed up as follows: decision-making on having an abortion on one’s own will to live a life on one’s own terms.

##### Actualized Her Intentions

Fourteen articles contained data regarding actualized young women intentions understood as a desire to be autonomous. Four articles found that young women had already made their abortion decision easily or had already decided by themselves. This indicates that they exercised their own will and were already certain about their decision [31,34,36,45].

Other findings were reported in six articles wherein adolescents felt that the final decision was an individual one, emphasizing their autonomy and having no regrets about their decision. They sought to have autonomy and control over their reproductive lives [29,35,37,41,42,45].

One study mentioned abortion positively as a solution to premarital pregnancies because men do not like women who have children [32]. Abortion is also possible for unintended pregnancies regardless of the circumstance or situation [44].

Four articles reported the decision of women to terminate their pregnancy to maintain their autonomy. They decided not to be influenced by others [29,30,33,38]. Some young women wanted to avoid dependency on men, thereby they worked toward their empowerment [30,38]:

“I don’t want to be dependent on somebody else… I want stability… and I don’t want to live off a guy either, on his salary, in his apartment!”

(Termination of Pregnancy [ToP], 17 years old, [30], p. 176)

##### Never Give up Education and Career

Twelve articles discussed the participants’ desire to “never give up education and career,” which is necessary for opening up their future. Bell et al. [36] examined the differences in decision-making between the termination of pregnancy (ToP) group and the continuation of pregnancy antenatal (AN) group. There were more who mentioned “career plans” as a factor in the ToP group than in the AN group. Furthermore, all those in the ToP group were unwilling to give up their plans:

“I am doing really well on the course at the minute, and I don’t want to give it up…”.

(ToP 16 years old, [36], p. 2508)

These findings are supported by other articles, indicating that the participants focused on their own future and own personal circumstances. Resorting to abortion helped them to actualize their dreams to secure a better future. Decision-making was influenced by the vision that they had of their own future. In this context, it is important to understand how young women prioritize self-realization [33,34,38,42,46].

Ramakuela et al. [39] found that pregnant learners opt for ToP because pregnancy at that age interferes with learning and teaching. Some schools did not allow pregnant learners to attend classes or required them to at least bring a parent to assist them in case of emergency. Being learners themselves, young women are concerned and aware of the lifelong effects of becoming pregnant. Additionally, seven other articles described the desire to continue education as a reason for their abortion decision-making [29,31,32,34,35,41,46].

#### 3.2.2. Unwanted Pregnancy

Fourteen articles reported on unwanted pregnancy that had three subcategories: dilemma and justification, not ready to be a mother, and worry about physical health. This category reported on a sudden life turning point wherein adolescents experienced feelings of confusion and conflict. This indicates young women’s attitude of ambivalence and their justification of abortion.

##### Dilemma and Justification

Eight articles reported about dilemma and justification. The decision to have an abortion is an emotional one. These women expressed a wide range of emotions because of their unexpected and unplanned pregnancies. Some young women are viewed negatively for what is often a mistake and can feel “attacked by everyone”. This emotion is related to their decision-making toward having an abortion [36].

Moreover, eight articles reported that pregnancy had been a shocking experience for most young women. They experienced emotional ambivalence when explaining their own decision-making [29,30,33,35,36,38,42,43]:

“It’s a horrible thing to do but I think it was the best thing”.

(ToP, 19 years old, [42], p. 9)

##### Not Ready to Be a Mother

Thirteen articles included the reason classified as not ready to be a mother. For unwanted pregnancies, personal factors were mostly mentioned such as too young or wrong timing and not ready to be a mother.

Some adolescents were aware that getting pregnant as a teen in high school was not proper [36,39]. The majority of these young women chose ToP and mentioned “stage of life” as a reason for choosing an abortion. These predicaments suggest that the teens were too young. The abovementioned findings are supported by previous studies [30,33,34,37,38,42].

Furthermore, most young women mentioned that they were not prepared to be mothers [29,30,31,33,36,38,40,42,43,46]. One of the young women stated that she is still immature:

“I didn’t think I was going to be a good mother because even myself, I still need some care and still need someone’s shoulder to cry on, especially my Mom”.

(ToP, adolescent, [29], p. 78)

##### Worry about Physical Health

Interestingly, there are two articles reporting on aspects related to worry about physical health. Bell et al. [36] indicated that an adolescent’s “own previous experience” of pregnancy contributed to her resolution and decision not to go through another unplanned pregnancy:

“I had loads of problems with pregnancy before … Last time I was pregnant my body was telling me I could cope. This time it didn’t sink in. My body was saying I am not ready”.

(ToP, 18 years old, [36], p. 2509)

One young women who was 15 years old rationalized her abortion in terms of her body being immature, as well as the challenge of her young age in carrying through the demands of a growing child [38].

Another young woman was worried about the damage and effects of her rapid delivery on her body:

“If you give birth in quick succession, your body will soon become like an ‘abrewa’ (old woman)”.

(ToP, 21 years old, [38], p. 926)

### 3.3. Interpersonal

Five categories were included under this domain: parental impact, reaction of partner, roles of peers and friends, existence of own child, and lack of support. These categories are described below with their associated subcategories.

#### 3.3.1. Parental Impact

All 18 studies included parental impact reasons for abortion. This category had three subcategories: pressure on making a choice regardless of her desire, fear of rejection and disappointment, and mediate the decisions.

Parental authority had a strong influence on young women who were in a vulnerable position. The following reactions were identified: fear of being kicked out of the house; fear of being rejected; worry, anger, and violence; and the desire not to disappoint their parents. This category summarizes the influence and reactions of parents who have a significant impact on the decision-making of their young vulnerable adolescent child. 

##### Pressure on Making a Choice Regardless of Her Desire

Sixteen articles indicated decision-making pressure from parents to have an abortion. Most of these cases were forced upon the young women regardless of their desire.

Some young women wanted to have an abortion but did not disclose this to their parents because they were concerned that their parents would disagree with their decision; others resorted to abortion because they were afraid of negative reactions such as being threatened, suffering violence, and experiencing anger from parents, and they wanted to avoid such reactions [29,30,31,32,33,35,37,38,39,40,41,42,43,46]. Botfield et al. [44] noted that views about adolescent abortion were not an acceptable topic or practice among the family and community if it involved premarital pregnancy. Young women are influenced by their parents’ reaction and attitude toward abortion.

Tatum et al. [33] reported two adolescents who experienced a traumatic event of being forced by their fathers to undergo abortion without their consent. There were also reports of cases where adolescents underwent secret unsafe abortions to avoid involving their parents:

“… wanted to do it quickly so that nothing [bad] happened and that [her] parents would not realize”.

(ToP, 16 years old, [33], p. 49)

Conversely, five articles indicated that despite the desire of adolescents not to interrupt their pregnancy, they reported that they had no autonomy to make such a decision, and they were forced to comply with their parent’ decision. Despite their late involvement, parents had a strong influence on the final decisions of adolescents [30,33,35,40,41]:

“I had an abortion against my own will”.

(ToP, 18 years old, [30], p. 176)

Domingos et al. [35] reported on abortion imposed by mothers. The mothers forced their daughters to drink special teas and take abortive medication in their own homes:

“My mother gave me ‘buchinha do norte’, tea pot, [and] cachaça prepared with cinnamon. None of these worked and I ended up in the hospital. […] I got pretty sick […] almost died” and continue she said […] It took a while for me to realize it. […] I felt guilty […]. Today, when I think about all I’ve done, I feel bad. […] I feel angry with my mom and with myself. I should have fought harder”.

(ToP, [35], p. 902)

Young women who experienced forced decision-making suffered and became frustrated for not having fought against their mothers’ decision, their lack of autonomy and power to decide about their preference, and the occasional guilt and regret as a result of an unsafe procedure [35,41].

##### Fear of Rejection and Disappointment

Eleven articles described fear of parental reactions, which was an important factor for making a decision to undergo abortion by the participants. Some studies indicated fear of being excluded from their family, damage to their relationships, and worry about losing support from their parents [29,31,32,37,40,42,44,45,46]. 

One young woman worried about rejection:

“As I am an orphan, and I live with my uncle, they are going to kick me out. No one would assist me”.

(ToP, 20 years old, [40], p. 7)

Interestingly, Bell et al. [36] noted that teenagers in the ToP group thought about the impact of abortion on their family more than in the AN group; no one in the AN group mentioned these factors. Similarly, three articles reported that young women considered the impact of abortion on their parents and they did not want to disappoint them. Abortion was also viewed as a potential solution to not disappointing their parents’ hopes [29,38,44]. Thus, some young women had to keep secrets from their parents including having to terminate their pregnancy [29,38]. 

One adolescent did not want to disappoint her parents:

“My parents would be angry. I didn’t want to disappoint them and I did [it] for them”.

(ToP, adolescent, [29], p. 78)

##### Mediate the Decisions

Seven articles reported on the parents’ attitude wherein they agreed with and assisted their young daughters in their choice. Young women sought emotional or decisional support from trusted parents or guardians in making a decision to undergo abortion [29,33,34,37,42,44,46].

One adolescent experienced unexpected support from parents:

“I never thought they would understand, but they did and I am grateful to them”.

(ToP, adolescent, [29], p. 75)

Additionally, young women needed and sought the assistance and accompaniment of an adult whom they trusted, someone including their parents to help them finalize and act on their decision [33,37]:

“I know she can help me… in making a decision about what to do”.

(ToP, adolescent, [37], p. 2209)

#### 3.3.2. Reaction of Partner

Seventeen articles showed the reactions of partners that explicate the influence of the partner’s reaction regarding abortion decision-making. The following three subcategories support this category: unstable relationship, negative and unhelpful, and consulted him but ignored her preference.

##### Unstable Relationship

Twelve articles indicated the impact of a young woman’s unstable partner relationship on her abortion decision-making. It was common for young women to worry about being abandoned or finding themselves actually abandoned if their partners discovered that they were pregnant. Occasionally, a partner left or broke up before a decision about the pregnancy had been made [29,31,32,33,34,40,41,42,43,45,46]. 

One of the young women stated:

“As soon as I told him [that] I was pregnant, he didn’t want to know. Haven’t seen him since”.

(ToP, 18 years old, [34], p. 10)

Tatum et al. [33] reported that in all the cases in which the partners were not involved, the respondents chose to terminate their pregnancy. This, however, may suggest that the absence of a partner may have encouraged the young woman’s autonomy. When one participant was asked whether she previously thought of herself as capable of making such an important decision by herself, she responded:

“No, I wanted my mother or [my boyfriend] to tell me what to do. When I saw that my cousin was supporting me in what I wanted [to do], when I saw [my boyfriend] with [another young woman], and when the doctor explained everything to me, then I was not afraid anymore”.

(ToP, 16 years old, [33], p. 48)

Ramakuela et al. [39] found that the majority of female teenagers with unplanned pregnancies did not live with their partners or have a committed relationship. These teenagers realized that they will be raising their child as single parents. Under such circumstance, most decided to have an abortion.

##### Negative and Unhelpful

Eleven articles contributed to this subcategory. Some articles reported that the partner’s denial and attitude of indifference can lead to ToP [29,30,31,32,33,38,39,40,43,46]. A young woman mentioned that the indifference shown to her by her partner influenced her decision to have an abortion:

“I took the final decision alone. When I told him about it, he wasn’t very happy and we quarreled. Though we are still together, he has not asked anything about it”.

(ToP, 22 years old, [38], p. 929)

Five studies reported on an unreliable and unhelpful partner [31,34,38,43,46]. For example, the partner was very young or was not involved as a partner for abortion decision-making. Oduro et al. [38] reported on a young woman who instead of informing and consulting her partner about her pregnancy, asked a friend for advice and acted on it because he would not have allowed the abortion:

“I did not tell the man who impregnated me, I know he would not have allowed the abortion”.

(ToP, 19 years old, [38], p. 929)

##### Consulted Him but Ignored Her Preference

This subcategory included data from 12 articles. In their study that examined the mindset of adolescents, Botfield et al. [44] mentioned the role of men as providing support to their female partners in their abortion decision-making process.

There were 11 articles indicating that young women consulted with their partners when a pregnancy was suspected or confirmed [29,30,33,34,35,37,38,40,43,45,46]. Although two of these articles reported that the male partners offered emotional support or commitment to help with raising the child, most of the articles reported that the male partners did not appear to support the preferences of the young women, and instead tried to convince them to abandon their initial preference to terminate their pregnancy. Thus, the male partners’ initial reaction was typically that young women should continue with their pregnancy, regardless of their preference. Nevertheless, several young women chose to have an abortion despite their partners’ offer of support [33,38]:

“[I asked him] what I should do. He told me, ‘Well, you should have it,’ and I told him he was crazy”.

(ToP, 15 years old, [33], p. 49)

In contrast, the responses of some male partners have been overwhelmingly negative toward continuing the pregnancy, with almost all supporting abortion. Ekstrand et al. [30], Frederico et al. [40], and Tatum et al. [33] described how the participants’ partners made implicit but clear remarks in favor of termination:

“He said, ‘Regardless of what you choose, I’ll support you—but you know what I’d prefer you to do…’ And of course, I wanted to respect that”.

(ToP, 18 years old, [30], p. 177)

#### 3.3.3. Roles of Peers and Friends

This section describes the roles of peers and friends. Eleven papers reported the influence of peers and friends on abortion decision-making. This category has one subcategory: supported decision-making.

##### Supported Decision-Making

Most of the articles reported that peers and friends are present, often as sympathizers, supporters, and counselors, although not exerting as much influence as the parents and partners [30,33,34,35,37,38,41,42,43,46]. Some young women initially shared the news with trusted peers or friends [33,35,37,38,43,46]. When young women were in need, their peers and friends provided various forms of physical support such as buying pregnancy tests, teaching them about emergency contraceptives, and providing information about abortion methods [33,46].

Moreover, Tatum et al. [33] mentioned that once the pregnancy was confirmed, peers and friends continued to provide emotional support by exploring options and helping respondents determine how they wanted to proceed. Notably, two articles reported that the opinions of friends did not appear to influence the participants’ final decision. In several cases, a decision was made that was opposite of their peers’ opinion about whether the participant should have an abortion or continue her pregnancy [33,41]. One study reported that adolescents decided to have an abortion for fear of losing friendship [39]. 

#### 3.3.4. Existence of Own Child

Young women who already had small children mentioned that the existence of their children was a major consideration in whether to keep their current pregnancy. This category has one subcategory: could not cope with another child.

##### Could Not Cope with Another Child

Five articles indicated that an influencing factor for the decision to have an abortion was the existence of one’s own child. Many of the young women stated that they could not cope with another child such as not being able to take care of another child. They also resorted to having an abortion for birth-spacing, considering the young ages of their children, and they also worried about the negative impact to their existing children [34,36,38,39,40]:

“I thought about my little girl. I don’t want to share my love with another baby”.

(ToP, 18 years old, [36], p. 2509)

#### 3.3.5. Lack of Support

Lack of support has one subcategory: desire emotional and physical support.

##### Desire Emotional and Physical Support

Eight articles stated that the lack of support from their parents, partners, and others influenced their choice of abortion. They expressed anger, sadness, and disappointment related to the lack of support from others [29,30,36,37,39,40,41]. Moreover, they needed not only physical support but also emotional support [29,37]. 

One adolescent girl sought emotional support from her mother:

“I am alone, that is the worst part. I didn’t have anyone to talk to except the people at the clinic and trusted that someone could help me, especially my mom”.

(ToP, adolescent, [29], p. 74)

### 3.4. Social Circumstances

Four categories were included under this domain: sexual crime, financial problem, limitation of choice, and underutilized healthcare services. These are described below along with their associated subcategories.

#### 3.4.1. Sexual Crime

This category has one subcategory: rape and incest.

##### Rape and Incest

Four articles indicated that rape and incest were related to abortion decision-making [39,40,45,46]. Some participants felt that teenage pregnancy from rape and incest would bring a curse and cause disruptions to the family [39]. These teens would rather opt for ToP instead of giving birth to a potentially disabled child in the family as a result of rape and incest. One adolescent said:

“I did it because I was raped by my uncle and my parents advised me to terminate [my] pregnancy because they felt that the baby will be a curse to the family and can also be disabled”.

(ToP, adolescent, [39], p. 4)

#### 3.4.2. Financial Problem

Financial problem has one subcategory: lack of financial autonomy.

##### Lack of Financial Autonomy

Fifteen articles found financial issues to be a major factor that influenced abortion decision-making. This included family financial status and the necessary financial stability. 

Ramakuela et al. [39] reported poverty as a major factor in making teenagers opt for ToP because they would not be able to take care of themselves and the child would become a burden to them and their families:

“I see poverty as a cause of terminating pregnancy due to poor family background wherein you will find that they depend on social grants of the grandparents”.

(ToP, adolescent, [39], p. 3)

Similar findings were reported in five other articles. Teenagers were concerned about adding another financial burden to their family, because most of them were financially dependent on their parents. Thus, a major factor influencing abortion decision-making was financial autonomy [32,33,35,40,41]. Additionally, eight articles reported that participants attributed abortion decision-making to financial difficulty and the necessity for stable finances [30,31,34,36,37,38,42,43,46]:

“I can’t give it anything, like I want to be able to have a kid when I’ve got money to bring it up”.

(ToP, 18 years old, [34], p. 10)

#### 3.4.3. Limitation of Choice

Twelve articles showed a limitation of choice. This category has three subcategories as stated and described below: fear of stigma, perspectives of religion, and misconception and lack of knowledge. 

##### Fear of Stigma

Nine articles pointed out the relevance of fear of pregnancy-related social impact and stigma in choosing ToP as a solution. Ramakuela et al. [39] stated that one of the main reasons teenage girls choose abortion is because they feared the stigma of being a pregnant teenager [30,32,36,38,39,41,46].

Some studies found that pregnancy outside marriage was described as highly shameful [31,32,41,44]:

“I was worried, I feared that people would laugh at me and say bad things about me”.

(ToP, adolescent, [32], p. 32)

Additionally, Botfield et al. [44] reported that young migrants and refugees in Sydney were afraid of the embarrassment to the family name. Several young women believed that a woman would be ostracized from her family or community if she became pregnant outside marriage. Therefore, participants held the strong belief that families might view a “secret” abortion as preferable to pregnancy outside marriage. 

Furthermore, Bell et al. [36] reported “negative discourses,” in which adolescent participants expressed a negative image of an unwed teenage mother. For example, an adolescent girl who had ToP did not want to be seen as….

“a typical teenager who’s got pregnant and doesn’t care about anything”.

(ToP, 16 years old, [36], p. 2510)

##### Perspectives of Religion

Five articles reported about the influence of religious perspectives on abortion decision-making. All articles showed that ToP was against the participants’ religious beliefs. Ekstrand et al. [30] reported the impact of the social network of religion on participants. Some young women had encountered openly expressed attitudes against the forthcoming abortion, such as harsh remarks or remarks about God’s punishment.

One of the girls said about her parents:

“Since my parents are immigrants and Muslims, it was totally out of the question for me to tell them about the pregnancy…. They would have turned me away if they’d known. I felt I was forced to choose between my family and my unborn child”.

(ToP, 18 years old, [30], p. 176)

Despite the prevailing religious beliefs, young women chose ToP [29,30,41,43,44]. Mpshe et al. [29] reported guilt as a result of going against their religious norms. 

These articles show that although they were religiously against abortion, this was not reflected in their decision to have an abortion, except to the extent that they felt guilty after having the abortion. This finding was also supported by Bain et al. [41], except that the situation about abortion was difficult to discuss openly.

##### Misconception and Lack of Knowledge

Ten articles touched on misconception and lack of knowledge regarding the law and abortion care. 

Three articles reported a lack of awareness about the legal status governing abortions. Even though ToP on certain grounds was legal, abortion seekers still viewed abortion as illegal [33,38,41]:

“I don’t know that such a law existed in Ghana”.

(ToP, 24 years old, [38], p. 925)

Nevertheless, Tatum et al. [33] mentioned that the legal status of abortion did not appear to be the main factor in deciding whether to continue the pregnancy or to seek termination services. Two young women who initially thought abortion was illegal, nonetheless, terminated their pregnancy [33]:

“I very much took that into account. I said, well, I do not think they can put me in jail”.

(ToP, 17 years old, [33], p. 51)

However, Bain et al. [41] pointed out that lack of awareness on existing legislation was a barrier to accessing available safe abortion services. 

Eight articles reported that there was not enough correct information regarding ToP. In this case, there was usually no open discussion about abortion; thus, young women had to make abortion decisions in the face of uncertain or insufficient information. Hence, they feared possible death or damage to some of their vital organs [29,30,31,32,33,39,44,46].

#### 3.4.4. Underutilized Healthcare Services

Thirteen articles reported on underutilized healthcare services, creating barriers to safe abortion procedures, and negatively affecting pregnancy outcomes. The importance of easy accessibility to abortion clinics was mentioned. This category has five subcategories: level of need for counseling, unaffordable, confidentiality and privacy issues, healthcare provider’s negative attitude, and accessibility barriers.

##### Level of Need for Counseling

Four articles described counseling. Two of these four articles indicated that many young women do not feel the need for pre-abortion counseling because they had already decided to terminate their pregnancy. Notably, they had already discussed ToP with another trusted person because of unreliable healthcare providers [33,34]:

“Because I didn’t feel I needed to, because I’ve talked a lot about it with my Mum, I talked about it with my boyfriend, I talked about it with my friend”.

(ToP, 18 years old, [34], p. 9)

However, the other two articles reported that counseling was effective. A few young women expressed appreciation for the neutral support of most healthcare providers and felt the need for professional counseling [29,30].

##### Unaffordable

Three articles reported that healthcare services were unaffordable, keeping these services from being utilized. This affordability problem can hinder access to safe abortion care [41,46]. These findings were supported by those of Frederico et al. [40] who reported that participants visited a health facility, but could not avail of the services because of their lack of money to pay for an abortion:

“They charged us money that we did not have. The ladies did not want to negotiate anything. I think they wanted 1200 mt (17.1 euros) if I am not wrong. He [boyfriend] had a job, but he did not have that amount of money”.

(ToP, 22 years old, [40], p. 8)

##### Confidentiality and Privacy Issues

Seven articles reported on confidentiality and privacy issues [30,31,32,40,41,45,46]. Most adolescents were afraid of healthcare providers’ disclosure to their relatives or community members [31,41,45,46]. In countries where abortion is illegal, there was worry that adolescents might be handed over to the police [32]. Chamanga et al. [32] indicated that this fear delayed the seeking of help by adolescents when they were in great pain and had developed complications following the unsafe induced abortion:

“I did not want to come to the hospital because I was worried, I was afraid that the nurses would report me to the police, and the police would arrest me”.

(ToP, adolescent, [32], p. 32)

##### Healthcare Provider’s Negative Attitude

Six articles described the negative attitude of healthcare providers. For some participants, the healthcare provider’s attitude was a deciding factor in whether to seek an abortion. These findings are also mentioned by Griffin et al. [46]. Three articles reported that young women were afraid of encountering negative attitudes of healthcare providers and concluded that such fear also contributed to the underutilization of health facilities [30,32,33,46]. Three articles noted the judgmental attitude of a physician specifically about age and the physician’s negative attitude [33,45,46]. Moreover, most participants were afraid to visit the hospital for unfounded fears of being shouted at or being hit by health workers [32]:

“I was worried because friends told me that people are beaten up when they go to the hospital. So, I was worried that they were going to slap me and add to the pain I was already feeling at the time. After coming here, I know these were lies, I was received and treated well here”.

(ToP, [32], p. 32)

On the other hand, the choice of medical services was facilitated when knowing a specialist in the healthcare facility [40]:

“I already knew who could induce it (abortion). No, I knew that person. I went to the hospital, and I talked to her, (and) she helped me”.

(ToP, 22 years old, [40], p. 8)

##### Accessibility Barriers

Nine articles reported several problems related to accessibility barriers of healthcare services. The first problem was the lack of information regarding abortions. Griffin et al. [46] indicated lack of access to accurate, up-to-date information about safe abortion options, their right to access services, and how to access them may be contributing to their nonuse. Mpshe et al. [29] found that some of the pregnancies of the participants were almost outside the terms of legal termination when they sought the abortion. There was also a lack of awareness or access to information on services that could provide appropriate information and care in their local areas [31,33,38,40,41,43,44,46]:

“I think it’s just the fact that a lot of young people don’t know they have access to that kind of stuff”.

(Nonpregnancy, 22 years old, [44], p. 204)

In addition, one article reported on a questionable health facility. Frederico et al. [40] described those treated at the hospital as saying that legal procedures were not followed and official receipts were not provided upon payment.

Owing to the lack of accessibility, Tatum et al. [33] reported that abortions were also performed outside of the health facility. Even with the involvement of an adult in the choice of place and method of pregnancy termination, this did not ensure the safety of the procedure [33].

## 4. Discussion

This scoping review identified 18 qualitative studies from 14 countries and included 1543 adolescents and young women participants. The findings were clustered into three domains: personal, interpersonal, and social circumstances. These three domains were supported by 11 categories and 24 subcategories including narratives of adolescents and young women regarding abortion decision-making. Bain et al. [47] explained that the Social Ecological Model assumes that behaviors and decisions are shaped by individuals, relationships, communities, and social factors. Similar studies used domains, akin to this study, that affected abortion decision-making, which included the following main factors: individual, others, and environment [38,40,48,49].

The first aim of this study was to characterize and map the factors that influence the abortion decision-making process of adolescents and young women. This narrative scoping review included all countries wherein all three domains were found to be relevant determinants of abortion without bias. This is likely related to the fact that the women are not financially autonomous and are in a vulnerable position as young people. Adolescent pregnancy is not the result of an intended choice; in addition, young women often have little say in the decisions that affect their lives [16]. Adolescent pregnancy is a global issue occurring in high-, middle-, and low-income countries [11].

### 4.1. Barriers to Autonomy of Decision-Making

In the present review, all articles provided data supporting the category of parental impact toward abortion decision-making. There have been several articles on abortion decision-making factors for young women, primarily on parental influence [50,51,52]. Some articles compared adults to minors in terms of decision-making toward pregnancy outcomes. The articles reported that minors were more likely to consider the effect of their pregnancy on their parents, with external factors (e.g., family pressure) precluding pregnancy continuation [20,52,53,54]. 

Regarding decision-making in dependent relationships (e.g., parent–child relationships), the feelings of guilt, shame, and loyalty appear to be more closely intertwined, and parental involvement in decision-making reflects dependence [55]. In this subcategory, the pressure of making a choice regardless of her desire comes with the fear of parental rejection and disappointment, as well as the need to mediate the decision. These findings indicate that complex emotions are intertwined in abortion decision-making, consistent with previous studies. 

In addition, autonomous decision-making is made with a sense of freedom; forced decision-making would be contrary to and a violation of this free choice, especially when there is a sense of pressure, control, and coercion [55]. The present review found pressure from parents to be a major factor influencing autonomous decision-making. When the decisions of adolescents are controlled by external forces, they are likely to experience emotional distress manifested as guilt and regret about their abortion more than those who went through the process of personally justifying their abortion decisions [52,53].

Some studies reported that unsafe abortion practices were a factor that made young women hide their pregnancies and abortion decision-making from their parents or guardians to avoid disappointment, resentment, and fear of stigma [20,56]. Thus, negative attitudes, such as pressure and fear, can lead young women to have an abortion even if the procedure used is unsafe. Gedan [57] mentioned that family counseling is viewed as especially important when abortion cannot be performed without a parent’s permission [57]. On the other hand, it also has some difficult aspects such as issues of adolescent’s autonomy and confidentiality regarding family, the male partner, and even local regulations [58]. However, Marecek [58] mentioned goals for counseling are to help the young woman obtain a final decision and to provide emotional support and referral information [58]. Therefore, we believe that it is important to confirm a young woman’s intention and consider counseling with someone who will influence the abortion decision-making, while ensuring confidentiality and ethical considerations.

Within the personal domain, the identified factors influencing abortion decision-making were self-realization and unwanted pregnancy. Each of these indicates that the decision to have an abortion is a spontaneous choice to be autonomous using one’s own reason. Grootens et al. [59] concluded that adolescents 12 years of age have the capacity to make adequate decisions given favorable environmental factors. However, adolescents’ decision-making depends on their abilities that vary across situations, and they may be able to make autonomous decisions with the support of facilitative environmental factors, such as having the right information, at the right time, in the right place, by the right person, and in the right way [59,60].

The present review found that adolescents needed and sought assistance and accompaniment from adults whom they trusted, including their parents, to finalize and then act on their decision [33,37]. Hoggart [42] indicated that if a woman is confident that she made the right decision and had no doubts or regrets, then the decision is comfortable.

Considering these findings, it is ultimately important that individuals be able to arrive at a point where they can feel confident enough to make an autonomous decision. To achieve this, people around them should be both involved in a noncoercive attitude and be supportive. To have an abortion in a safe, secure, and appropriate environment, it is necessary to enhance abortion decision support, provide correct knowledge, and give accurate information for adolescents to make an informed choice. 

Some studies of decision aids suggest that these aids improve patient decision-making compared with the usual care [61,62]. At the same time, the reported decision aids were poorly used in daily practice [61]. Presently, research on abortion decision-making aids remains insufficient [63]. There is therefore an urgent need to promote the use of abortion decision-making aids.

The present review found that the identified role of the partner changed depending on the situation and relationship status. Some articles reported the influence of partners [64,65]. However, in the present review, some articles reported that the partners did not influence the final decision-making [33,41]. In a review of adolescent men’s attitudes and decision-making regarding pregnancy outcome, it was reported that the findings of studies can often be contradictory owing to their “…attitudes about unintended pregnancy, contraception, and abortion and can be contextual and contingent on the dominant or prevailing social norms and roles within a given time, space or social group” ([66], abstract, results section). Thus, the findings of this present research coincide with those of previous research.

### 4.2. Barriers to Accessibility of Healthcare Services

The second aim of this study was to identify the care and support needed by adolescents in their decision-making process. The first need is to promote decision-making aids so that adolescents can make informed decisions, and the second need is to increase the use of healthcare services. However, the underutilized healthcare services were found to be an important category. Adding strength to this category are the subcategories level of need for counseling, unaffordable, confidentiality and privacy issues, healthcare provider’s negative attitude, and accessibility barriers.

Because of personal issues, and the interpersonal and social pressures associated with ToP, the decision to have an abortion may involve conflict [67]. Nevertheless, adolescents may not have used healthcare services because they were anxious, had fear of unfounded rumors, lacked knowledge, held the negative attitudes of healthcare providers, feared disclosure of secrets, and found abortion unaffordable [4,7,49]. The negative effects of this include unsafe abortion practice and delays in seeking medical attention in case of complications [19,20,56].

The present review clarified that most of the women had already discussed about seeking an abortion or viewed the issue as very private so that they carefully chose the person(s) they talked to. Therefore, pre-abortion counselling was helpful only for some women and it was viewed as unnecessary by most women. The abortion decision-making process varies with individual differences. Women who want to have an abortion are often already sure of their decision and do not need counseling, whereas women who are not yet sure of their decision likely need and should be offered suitable counseling [68,69]. Because there was a lack of information about the induce abortion procedure [33,34], it will be necessary to consider how to provide effective counseling that meets the needs of the target population regardless of their decision status.

Additionally, many studies have mentioned the negative attitudes of and the stigma from healthcare providers [20,49,65,70]. These attitudes and stigma act as barriers to access healthcare services. Interestingly, the choice of medical services is strengthened by knowing someone in the health facility [40]. Thus, it is important for healthcare providers to be friendly and reliable [71]. Adolescent and youth-friendly health services are aimed at improving the quality of healthcare services and increasing access to reproductive care services for young people [72,73,74]. The WHO has produced a guidebook called “Making Health Services Adolescent Friendly” that provides the public health rationale for making sexual and reproductive health services more accessible to adolescents. In it, they define “youth-friendly health services” from a quality perspective and provide step-by-step guidance on the development of quality standards for the provision of health services to adolescents [72]. Furthermore, the youth health services training resource package, developed by UNFPA in collaboration with YP Foundation, is designed for healthcare providers. Its purpose is to enable them to understand the common sexual and reproductive health (SRH) needs of young people, and to help them build their skills to provide respectful, confidential, and nonjudgmental SRH services to young people [74]. This underscores the importance of training healthcare providers on how to provide adolescent and youth-friendly health services. 

Finally, in the present review, some adolescents and young women experienced feelings of guilt and regret after having an abortion, indicating the need for post-abortion counseling [29,32], particularly because the results of an unsafe abortion may cause post-abortion complications [5,20,75]. In this way, healthcare services can be involved in both physical and mental care before, during, and after the abortion.

## 5. Limitations and Strengths

In terms of limitations, this scoping review included only 18 articles because this study’s focus was on the examination and comparisons of findings of qualitative articles written in English. Furthermore, this scoping review was restricted to participants aged 10–24 years. Some studies included not only young women who had an abortion but also young women who were pregnant or not pregnant. In addition, although the research was conducted without limiting the area, there was a bias in the area. Thus, some themes that are characteristic of each age group may have been missed, making the results nongeneralizable to all settings. This narrative scoping review explores decision-making factors and does not focus on specific decision-making methods, types of measures, or frequency of use. We plan to utilize this information in our next research to support decision-making.

In terms of strengths, this scoping review is, to the best of our knowledge, the first narrative scoping review that examined the abortion decision-making factors of adolescents and young women. This scoping review was also not limited to certain countries. Moreover, the findings showed that the abortion decision-making process of adolescents and young women is influenced by various personal, interpersonal, and social circumstances, regardless of the country’s socioeconomic status: high-, middle-, or low-income countries. Finally, the categories were robustly supported by subcategories; thus, this scoping review could be used for mapping. The identification of important factors influencing abortion decision-making of adolescents and young women in this narrative scoping review is anticipated to contribute to the development of a decision-support aid in the near future based on the real voices of women as identified in this narrative review.

## 6. Conclusions

The abortion decision-making process of adolescents and young women is influenced by various personal, interpersonal, and social circumstances. In particular, young women who are dependent on their parents for their livelihood are often left in a vulnerable position. This situation is the same in every country regardless of the country’s socioeconomic status (i.e., high-, middle-, or low-income status). Thus, parents are especially influential and tend to force their daughters to make a decision often against their will. Unwanted pregnancy causes a sudden life turning point, causing confusion and conflict among adolescents and young women. They often experience suffering and frustration for not standing up against their parents’ decision, as well as for their lack of autonomy and power to decide in accordance with their preference. Adolescents and young women usually desire autonomy in decision-making. Therefore, it is necessary for those around these still-immature women to provide them support and to encourage their autonomous decision-making by providing appropriate and sufficient information. 

Notably, healthcare services related to abortion decision-making remain underutilized. To improve access to these healthcare services, the following items must be implemented: (1) training of staff to provide adolescent and youth-friendly health services; (2) counseling of adolescents and young women based on their needs, and counseling involving their parents or guardians while ensuring confidentiality and ethical considerations; (3) promotion of decision aids; and (4) care that is affordable and accessible, so that pregnant adolescents and young women can take full advantage of healthcare services.

## Figures and Tables

**Table 1:** Inclusion and exclusion criteria based on the population, concept, and context framework.

PCC Framework	Inclusion Criteria	Exclusion Criteria
Population	Adolescents aged 10–19 years Young women aged 10–24 years (Defined by WHO [26])	
Concept	Research factor of decision-making abortion Influence the choice behavior of pregnancy outcome Research view of abortion Qualitative study, mixed methods narrative story, interview, diary	Research examining miscarriage, abortion of fetus Research examining abortion procedures and option Research examining abortion of biased target Research examining adolescent sexual behaviors or activities, and contraception Research examining the effectiveness of interventions or pregnancy prevention programs
Context	Any institution, health facility, and country	
Language	English	Non-English
Publication type	Original studies Primary research articles	Only abstracts Conference proceedings Trial registrations Reviews (e.g., scoping review, systematic review)
Publication date	All articles published on the database up to October 2023	

**Table 2:** Research findings displayed by domains, categories, and subcategories.

Domains	Category	Subcategory	Mpshe et al., 2002 [29]	Ekstrand et al., 2009 [30]	Jejeebhoy et al., 2010 [31]	Chamanga et al., 2012 [32]	Tatum et al., 2012 [33]	Brown S., 2013 [34]	Domingos et al., 2013 [35]	Bell et al., 2014 [36]	Hasselbacher et al., 2014 [37]	Oduro et al., 2014 [38]	Ramakuela et al., 2016 [39]	Frederico et al., 2018 [40]	Bain et al., 2019 [41]	Hoggart L. 2019 [42]	Ouedraogo et al., 2020 [43]	Botfield et al., 2020 [44]	Chiweshe et al., 2021 [45]	Griffin et al., 2023 [46]
Personal	Desire for self-realization	Actualized her intentions	✓	✓	✓	✓	✓	✓	✓	✓	✓	✓			✓	✓		✓	✓	
		Never give up education and career	✓		✓	✓	✓	✓	✓	✓		✓	✓		✓	✓				✓
	Unwanted pregnancy	Dilemma and justification	✓	✓			✓		✓	✓		✓				✓	✓			
		Not ready to be a mother	✓	✓	✓		✓	✓		✓	✓	✓	✓	✓		✓	✓			✓
		Worry about physical health								✓		✓								
Interpersonal	Parental impact	Pressure on making a choice regardless of her desire	✓	✓	✓	✓	✓		✓		✓	✓	✓	✓	✓	✓	✓	✓	✓	✓
		Fear of rejection and disappointment	✓		✓	✓				✓	✓	✓		✓		✓		✓	✓	✓
		Mediate the decisions	✓				✓	✓			✓					✓		✓		✓
	Reaction of partner	Unstable relationship	✓		✓	✓	✓	✓					✓	✓	✓	✓	✓		✓	✓
		Negative and unhelpful	✓	✓	✓	✓	✓	✓				✓	✓	✓			✓			✓
		Consulted him but ignored her preference	✓	✓			✓	✓	✓		✓	✓		✓			✓	✓	✓	✓
	Roles of peers and friends	Supported decision-making		✓			✓	✓	✓		✓	✓	✓		✓	✓	✓			✓
	Existence of own child	Could not cope with another child						✓		✓		✓	✓	✓						
	Lack of support	Desire emotional and physical support	✓	✓		✓				✓	✓		✓	✓	✓					
Social circumstances	Sexual crime	Rape and incest											✓	✓					✓	✓
	Financial problem	Lack of financial autonomy		✓	✓	✓	✓	✓	✓	✓	✓	✓	✓	✓	✓	✓	✓			✓
	Limitation of choice	Fear of stigma		✓	✓	✓				✓		✓	✓		✓			✓		✓
		Perspectives of religion	✓	✓											✓		✓	✓		
		Misconception and lack of knowledge	✓	✓	✓	✓	✓					✓	✓		✓			✓		✓
	Underutilized healthcare services	Level of need for counseling	✓	✓			✓	✓												
		Unaffordable												✓	✓					✓
		Confidentiality and privacy issues		✓	✓	✓								✓	✓				✓	✓
		Healthcare provider negative attitude		✓		✓	✓							✓					✓	✓
		Accessibility barriers	✓		✓		✓					✓		✓	✓		✓	✓		✓

Note: The symbol ✓ means applicable.

## Data Availability

All data generated or analyzed during this study are included in this published article.

## Abbreviations

AN	Antenatal
AYFHS	Adolescent and Youth-Friendly Health Services
CASP	Critical Appraisals Skills Programme
JBI	Joanna Briggs Institute
PRISMA-ScR	Preferred Reporting Items for Systematics reviews and Meta-Analyses extension for Scoping Reviews
SRH	Sexual and reproductive health
ToP	Termination of pregnancy
WHO	World Health Organization

## Appendix A

**Table A1:** Search strategy.

#1	“adolescent pregnancy” or “pregnancy in adolescence”
#2	“adolescent” or “teenage” or “teenager” or “young women” or “youth”
#3	#1 and #2
#4	“abortion” or “induced abortion” or “termination pregnancy”
#5	“decision making” or “choice behavior” or “determinants” or “factor”
#6	#3 and #4 and #5
#7	“qualitative” or “qualitative study” or “mixed method”
#8	#6 and #7

## Appendix B

**Table A2:** Excluded articles.

Articles	Reason for Exclusion
Lema et al., 1996 [76]	The aim of the study is different (i.e., assessment of unsafe abortion)
Finer et al., 2005 [77]	The participants are over 24 years of age.
Kapil et al., 2005 [78]	There is no interview raw data
Helström et al., 2006 [79]	There is no interview raw data
Ratlabala et al., 2007 [80]	The aim of the study is different (i.e., understanding perception and constraints in accessing ToP services)
Hess RF. 2007 [81]	The participants are over 24 years of age
Puri et al., 2007 [82]	All the participants are married
Olsson et al. 2010 [51]	The participants are over 24 years of age
Biggs et al., 2013 [83]	The participants are over 24 years of age
Kumi-Kyereme et al., 2014 [84]	The participants are over 24 years of age
Appiah-Agyekum et al., 2015 [85]	There are no interview raw data and the participants were over 24 years of age
Aziato et al., 2016 [86]	The study design was different (i.e., a vignette-based focus group approach)
DePiñeres et al., 2017 [87]	The aim of the study was different (i.e., seeking safe and legal services)
Kebede, M. T. et al., 2018 [88]	The aim of the study was different (i.e., search of abortion services)
Oyeniran, A. A. et al., 2019 [89]	The participants are out of target age
Baum, S. E. et al., 2020 [90]	The participants are out of target age
Bain et al., 2020 [47]	The aim of the study was different (i.e., stakeholders’ and adolescents’ views of early pregnancy)
Chareka, S. et al., 2021 [91]	The aim of study was different (i.e., focus young women who sell sex)
Ituarte et al., 2021 [92]	The article is not in English
Chainok L. et al., 2022 [93]	The aim of study was different (i.e., search of undergoing legal abortion)
